# Association between time-weighted activity space-based exposures to fast food outlets and fast food consumption among young adults in urban Canada

**DOI:** 10.1186/s12966-020-00967-y

**Published:** 2020-05-13

**Authors:** Bochu Liu, Michael Widener, Thomas Burgoine, David Hammond

**Affiliations:** 1 Department of Geography and Planning, University of Toronto, 100 St. George Street, Toronto, ON M5S 3G3 Canada; 2 UKCRC Centre for Diet and Activity Research (CEDAR), MRC Epidemiology Unit, University of Cambridge School of Clinical Medicine, Box 285 Institute of Metabolic Science, Cambridge Biomedical Campus, Cambridge, CB2 0QQ UK; 3 School of Public Health and Health Systems, University of Waterloo, 200 University Ave W, Waterloo, ON N2L 3G1 Canada

**Keywords:** Fast food outlet exposure, Fast food consumption, Young adults, Time weighting, Activity space, Canada food study

## Abstract

**Background:**

Despite increased attention on retail food environments and fast food consumption, results from previous studies have been inconsistent. Variation in measurement of exposure to retail food environments and the context of the built environment are possible reasons for inconsistencies. The purpose of the current study is to examine the association between exposure to fast food environment and fast food consumption among young adults, and to explore possible associations between built environment and fast food consumption.

**Methods:**

We employed an observational, cross-sectional study design. Cross-sectional surveys were conducted in 2016 and 2017. In a sample of 591 young adults aged 16–30 years in five Canadian cities, we constructed and computed individual-level time-weighted number and ratio of fast food outlets in activity spaces derived from GPS trajectory data. Negative binomial regression models estimated the associations between exposure measures and frequency of fast food consumption (number of times consuming fast food meals in a seven-day period), controlling for built environment characterization and individual-level characteristics.

**Results:**

Significant positive associations were found between time-weighted *number* of fast food outlets and count of fast food meals consumed per week in models using a radius of 500 m (IRR = 1.078, 95% CI: 0.999, 1.163), 1 km (IRR = 1.135, 95% CI: 1.024, 1.259), or 1.5 km (IRR = 1.138, 95% CI: 1.004, 1.289) around GPS tracks, when generating activity spaces. However, time-weighted *ratio* of fast food outlets was only significantly associated with count of fast food meals consumed when a radius of 500 m is used (IRR = 1.478, 95% CI: 1.032, 2.123). The time-weighted Active Living Environment Index with Transit measure was significantly negatively related to count of fast food meals consumed across all models.

**Conclusions:**

Our study demonstrated associations of time-weighted activity space-based exposure to fast food outlets and fast food consumption frequency in a sample of young adults in urban Canada, and provides evidence of the association between context of built environment and fast food consumption, furthering discussion on the utility of individual-level, activity space-based data and methods in food environment research. These results imply that both food retail composition and activity spaces in urban areas are important factors to consider when studying diets.

## Background

Fast food consumption is an established risk factor for overweight and obesity [1], and increased patronage of fast food outlets has been associated with weight gain over time [2, 3]. To understand the population-level determinants of fast food consumption, researchers have actively explored linkages between neighborhood fast food exposure and fast food dietary behaviors [4]. Researchers have examined the links between levels of fast food consumption and exposure to fast food outlets with a focus on student [5], adolescent [6], middle-aged [7], and entire population groups [8, 9].

Despite researchers’ increased attention on retail food environments and fast food consumption, results from studies have been inconsistent. Some have identified associations between exposure to fast food outlets in residential and school neighborhoods and fast food consumption [9–13], while others have found counterintuitive associations [14] or no association [15]. Apart from differences in populations, study areas, and study periods, which may lead to varied results, both differences in how exposure to the retail food environment has been measured and how the context of the built environment affects a person’s interactions with their surroundings are possible reasons for the aforementioned inconsistencies.

### Measuring the food environment

Many approaches taken by researchers to measuring food environment exposure have been subject to the Uncertain Geographic Context Problem (UGCoP). This problem results from spatial uncertainty in identifying the truly relevant geographic area that exerts behavioural influence, and the temporal uncertainty in the timing and duration in which individuals experience these contexts [16]. This problem arises in part from data availability, but also from an inability to define causally relevant geographic contexts for many behaviors of interest [17]. For example, because fast food consumption occurs beyond home or school neighborhoods, within which fast food outlet exposure is often measured, it is likely necessary to measure fast food outlet exposure in broader ‘activity spaces’ [18–20]. Activity spaces have been defined as “the local areas within which people move or travel in the course of their daily activities” [21]. To better account for human mobility and to measure activity spaces, Global Positioning System (GPS) tracking technology is increasingly used [18].

Despite this move to a more comprehensive consideration of study participants’ daily movement patterns and associated environmental exposures, few researchers have considered the duration of exposure in these spaces [18, 22]. Exceptions include work by Sadler and colleagues [6], in which adolescents’ time spent within 50 m of unhealthy food outlets between home and school was found to have a significant effect on the likelihood of purchasing junk food. Aiming to address the utility of a time-based measure of exposure to the food environment, a more methodologically-focused study found that the count of fast food restaurants study participants were exposed to was not associated with fast food restaurant visits. However, time-weighted counts of nearby fast food restaurants, where counts of fast food restaurants are weighted by time durations in proximity to these restaurants, were associated with significantly higher odds of fast food restaurant visits [23].

Another perspective on defining the appropriate measure of food environment exposure lies in the choice of using absolute or relative measures. Absolute measures such as of proximity, density, or count are more straightforward to interpret and have been widely used in studies employing activity space-based measures of exposure to the food environment [6, 12, 23]. Relative measures, for example defined as the proportion of food outlets of interest over the total number of food outlets available, adjust for wider food environment context by considering exposure to competing food outlets [17, 24]. Studies have argued that relative exposure measures better capture environmental risks for poor diet [25] and more consistently predict dietary behavior [26], however no definitive consensus has yet been reached on which type of measure is more appropriate.

### The effects of the built environment on dietary behaviours

Detailed characterizations of the built environment could be important to consider in order to control for how various urban forms may affect participants’ dietary behaviour and to provide geographic contexts when absolute and relative measures of exposure to fast food outlets are used. While it is known that characteristics of the built environment, such as density of urban amenities, can impact a range of behaviours (e.g. choice of transportation mode [27]), little work has been done to control for these characteristics in analyses of links between food environment exposures and dietary behaviors [28]. To account for this, some past studies have used basic area-level variables to distinguish between urban, suburban, and rural areas [9, 19, 29], or measures of distance to urban regions [10]. However, to this point, food environment researchers have not directly controlled for detailed characterization of built environment, which may associate with ways people use food outlets.

### Study approach

To address the gaps noted above, this study, which is focused on an understudied population of young adults (aged 16–30 years) in five urban regions of Canada, aims to construct both absolute and relative, time-weighted, activity space-based exposure measures of the fast food environment and to examine the association between exposure to the fast food environment and fast food consumption in this group.

Accomplishing these aims will provide both new insights into how the food environment affects dietary behaviours, as well as furthering discussion on the utility of individual-level, activity space-based data and methods in food environment research.

## Method

### Data

Data come from the Canada Food Study (CFS), which focused on young adults (aged 16 to 30 years) living in the Canadian cities of Toronto, Montreal, Vancouver, Edmonton, and Halifax [30, 31]. The CFS included a detailed lifestyle questionnaire and GPS data, collected using a version of the Itinerum smartphone application [32], from two survey waves in 2016 and 2017. The main survey with the entire CFS cohort was conducted in 2016 (hereafter, ‘Wave 2016’) with 3000 respondents, 1022 of which were successfully retained in 2017 survey (‘Wave 2017’), with a follow-up rate of 34.1% [31]. A sub-sample of CFS cohort members were recruited for the GPS Survey. Both the main surveys and GPS data in 2016 and 2017 were collected between October and December, and they did not overlap with school vacations or other long holidays. GPS data were collected over seven consecutive days for a subsample of 630 respondents in Wave 2016 and 400 in Wave 2017. For this analysis, respondents who participated in the GPS Survey for less than 72 h, as judged by the length of time the smartphone application was installed, were excluded, resulting in a study sample of 575 participants in 2016 and 373 in 2017. For those respondents who participated in both waves of GPS data collection, the wave with the longer recorded time period, in hours, of GPS data was retained, which resulted in a sample of 728 participants. A further 137 individuals were excluded due to missing data in other relevant variables. The final analytical sample included 591 participants, of which, 356 were from Wave 2016 and 235 from Wave 2017 (see Additional file 1 for more information about the sample).

In the final analytical sample, respondents were approximately equally distributed across Toronto, Vancouver and Halifax, with fewer respondents from Montreal and Edmonton. Female respondents accounted for nearly two thirds of the final study sample (data shown in “Covariates” section). Kolmogorov-Smirnov tests and z-tests for proportions are administered to ensure the data used in this paper are representative of the broader population who took the main survey. All variable distributions, with the exception of those derived from sex, age and residential city of Montreal, were not statistically different from those found in the main survey. When compared to the main survey, the GPS subset analyzed in this paper has a significantly higher proportion of females (subset: 65.0% vs. main: 60.5%, *p* < 0.05) and respondents aged between 25 and 27 years old (subset: 19.6% vs. main: 14.7%, p < 0.05), and a significantly lower proportion of respondents who are 16 to 18 years old (subset: 17.1% vs. main: 24.4%, p < 0.05) and who reside in Montreal (subset: 13.2% vs. main: 18.7%, p < 0.05).

### Outcome - frequency of fast food consumption

**Fig. 1:**
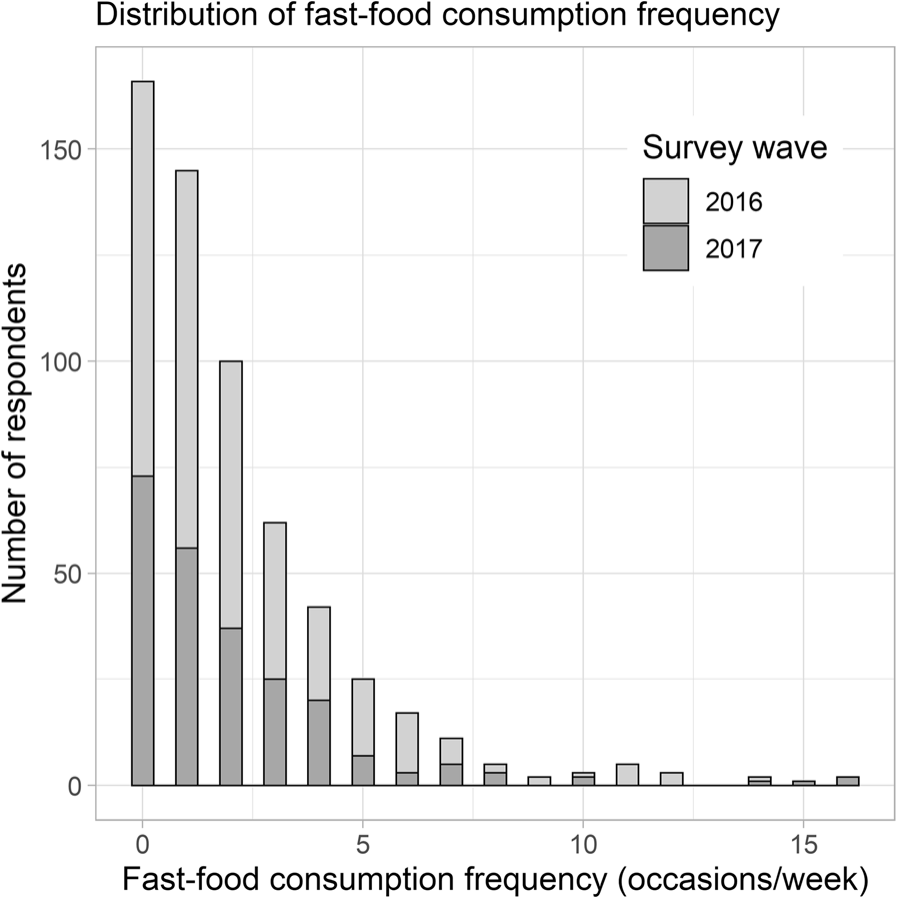
Distribution of count of weekly fast food consumption in Waves 2016 and 2017

The outcome variable of interest in this paper is frequency of fast food consumption, an observed count variable defined as the number of times respondents reported consuming fast-food meals over a week period. In the CFS questionnaire, respondents indicated where each meal prepared outside the home was purchased over a seven-day period, and responses of “Fast food / quick service / coffee shop (i.e., order from a counter, pizza delivery, etc.)” were considered to be of fast food. Weekly counts of fast food consumption ranged from 0 to 16 with a mean of 2.1, a median of 1.0, and a strong right skew (Fig. 1).

### Exposures to fast food outlets

GPS data were collected using Itinerum software [32], an open smartphone travel survey application. To generate activity spaces, the raw GPS tracking points were fed into an algorithm that identified activity locations at which a respondent spent at least 10 min per visit [33]. The reason activity locations are used to understand exposure instead of examining raw GPS trajectories is that the aim of this paper is to develop and explore a measure of cumulative exposure that represents habitual exposure to the food environment over a relatively longer time period. This measure not only represents the availability of fast food outlets in respondents’ activity spaces, but also captures the potential influence of these restaurants on respondents’ desire to eat, perceptions of what types of food are socially appropriate, and habitual patterns of food consumption [34]. The choice of the threshold of duration used to identify activity locations should enable the extraction of dwell points, while accounting for a reasonable amount of time spent stationary during the day. The sum of time spent in activity locations using the 10 min threshold selected for this study, on average, accounts for 93% of respondents’ total time of GPS tracking and the rest of time were likely spent during trips or short activities that last less than 10 min. In simpler terms, this equates to 22.3 h a day in stationary locations and 1.7 h making trips between locations. Details on the methods can be found on the GitHub page included in the references [33]. Briefly, this algorithm first removed points with a high probability of being an error, then computed a time-weighted Kernel Density Estimate (KDE) on the remaining data points. Next, a spatial-temporal linear interpolation was calculated when necessary, and finally activity locations with the assigned minimum activity duration were extracted. The activity locations derived from the algorithm are linked to every study participant, and include latitude, longitude, and time spent at each location. Three versions of activity spaces were created by buffering around each activity location with radii of 500, 1000, and 1500 m using GeoPandas 0.5.1 for Python [35]. These distances were commensurate with the radii used in studies on activity space-based measures derived with GPS data [12, 19, 36, 37] and will be used for sensitivity analysis.

To calculate absolute and relative measures of fast food outlet exposure within activity spaces, we used OpenStreetMap (OSM) data, which contains the locations of all food establishments within the study areas. OSM is an open-sourced collaborative platform for mappers to create a free editable map where everyone can access points of interest, road networks and other geographical information. OSM maintains a map between its classification scheme and the North American Industry Classification System (NAICS). Queries of “fast_food”, “supermarket”, “green_grocer”, and “convenience” in OSM correspond with Limited-Service Restaurants, Supermarkets and Other Grocery (except Convenience) Stores, Fruit and Vegetable Markets, and Convenience Stores respectively in NAICS [38]. All point features classified as fast food outlets, supermarkets, green groceries, and convenience stores in Census Metropolitan Areas (CMAs) of five urban regions were queried and stored. After mapping the food retail establishments and activity spaces using a common projection, the number of fast food outlets in each activity space was summed and linked to each participant as their absolute measure of fast food outlet exposure. Similarly, the number of fast food outlets as a proportion of the sum of the number of fast food outlets, supermarkets, green groceries, and convenience stores within each activity space, was calculated as the relative measure of fast food outlet exposure for each participant.

Finally, based upon the absolute and relative measures of fast food outlet exposure for each individual’s activity space, time-weighted measures of exposure were computed. These measures weight the exposures within activity spaces of an individual to fast food outlets, by the proportion of time spent in these activity spaces, relative to the total time spent across all activity spaces. Thus, the time-weighted count of fast food outlets in activity spaces is:

1 $$ {AE}_i=\sum \limits_{j=1}^{m_i}\frac{T_{ij}}{T_i}{F}_{ij} $$

and the time-weighted ratio of fast food outlets in activity spaces is:

2 $$ {R}_{ij}=\frac{F_{ij}}{S_{ij}+{G}_{ij}+{F}_{ij}+{C}_{ij}} $$

3 $$ {RE}_i=\sum \limits_{j=1}^{m_i}\frac{T_{ij}}{T_i}{R}_{ij} $$

where *AE*_*i*_ and *RE*_*i*_ are absolute and relative measures, respectively, of time-weighted exposure to fast food outlets for individual *i*; *m*_*i*_ is total number of activity spaces of individual *i*; *T*_*ij*_ is time spent in activity space *j* of participant *i*; *T*_*i*_ is total time spent in all activities spaces of individual *i*; *F*_*ij*_ is number of fast food outlets in activity space *j* of individual *i*; *R*_*ij*_ is proportion of fast food outlets in activity space *j* of individual *i*. *S*_*ij*_, *G*_*ij*_, and *C*_*ij*_ refer to the number of supermarkets, green groceries, and convenience stores, respectively, for activity space *j* of individual *i*.

**Fig. 2:**
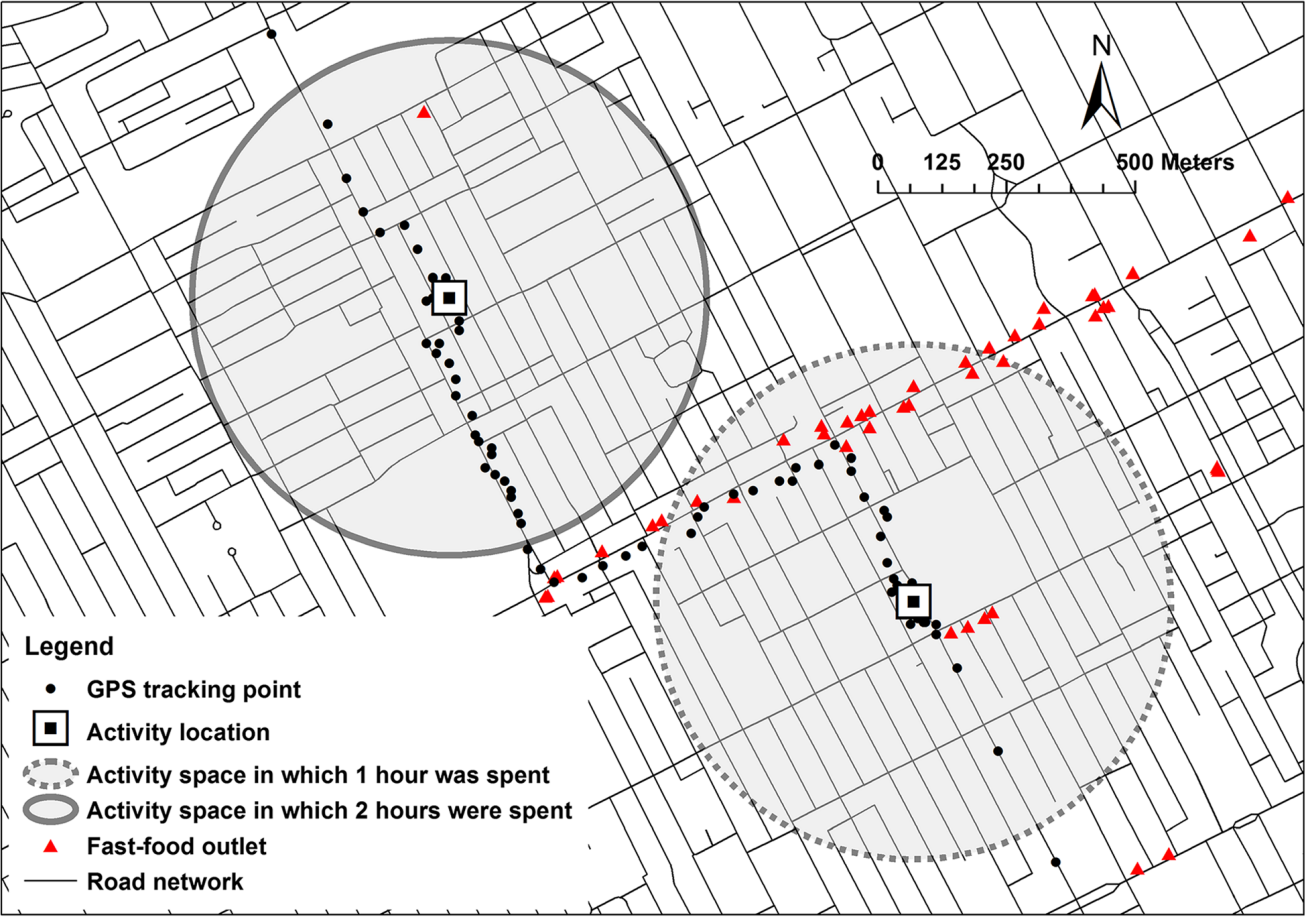
Illustration of time-weighted container approach. Note: GPS points were created by the authors for the purpose of illustration and were not a segment of any GPS trajectories of respondents

Figure 2 illustrates the elements we used to measure fast food exposure. Activity locations where an individual spent at least 10 min were identified, with the duration of activity recorded. Activity spaces were created by buffering the activity locations by a radius, as described previously. These activity spaces were the spatial containers used for quantifying both the number and the ratio of fast food outlets. Finally, the activity space-level indicators were weighted by the proportion of time spent in these activity spaces, relative to the total time spent across all activity spaces, and aggregated to an individual-level exposure measure. It is worth noting that activity space overlap does not alter the way to compute the time-weighted exposure. The exposures derived from the activity spaces, whether there is an overlap or not, are summed according the Formula (1) or (3).

Log transformations were applied to the heavily right-skewed variable of the time-weighted number of fast food outlets, so larger values were dragged down. This transformation is justified as there are likely to be diminishing effects of fast food outlet exposure as the number of retail opportunities reaches higher numbers. For four respondents who had activity spaces with zero fast food retailers, the minimum non-zero time-weighted number of fast food outlets was manually assigned before log transformation was performed to eliminate errors generated due to attempting to calculate the logarithm of zero. The time-weighted ratio of fast food outlets did not require transformation, given the total number of food retailers scaled with the number of fast food outlets. Summary statistics for measures of fast food outlet exposure are shown in Table 1.

**Table 1:** Summary statistics for measures of fast food outlet exposure

Variable	Spatial unit	Mean	S.D.	Minimum	P25	Median	P75	Maximum
**Time-weighted ratio of fast food outlets**	500 m buffer	0.383	0.270	0.000	0.144	0.349	0.601	1.000
	1 km buffer	0.492	0.219	0.000	0.358	0.520	0.648	1.000
	1.5 km buffer	0.540	0.180	0.000	0.453	0.561	0.663	0.989
**Log-transformed time-weighted number of fast food outlets**	500 m buffer	0.778	1.626	−5.020	−0.027	0.905	1.846	4.488
	1 km buffer	2.036	1.429	−3.609	1.232	2.106	2.852	5.531
	1.5 km buffer	2.747	1.307	−3.014	1.919	2.755	3.489	6.027

### Covariates

A variable describing the characteristics of the built environments of activity spaces and dummy variables indicating residential cities (Toronto as the reference) are included in the models to adjust for the different urban forms present in the five study cities. For the former, we use the “Active Living Environment Index with Transit” measure (hereafter, ALE measure) from the Canadian Active Living Environments Database (Can-ALE) 2016 [39]. This measure represents how dense the urban built environment is by computing the sum of the z-scores of intersection density, dwelling density, number of points of interest, and count of public transit stops within one-kilometre buffers drawn from the centroids of Dissemination Areas (DAs) across CMAs in Canada (See Additional file 2 for descriptions of the geographic units). The value for the ALE measure for a certain DA is determined by its position in the distribution of the index relative to all DAs in CMAs across Canada, which ensures the comparability of this metric across the five study cities. The ALE measure for each participant is computed by weighting the ALE measure for each activity space by the ratio of duration of the activity space to the total time spent in all activity spaces, which also corresponds to time-weighting approach that we used for the measures of fast food exposure.

Age and sex are adjusted for in the models. The perceived income adequacy variable is derived from a question on the self-reported difficulty to make ends meet, and was categorized in five levels from “very difficult” to “very easy”. It is selected as a socio-economic measure of income in this study because the traditional socio-economic measures of income are difficult to assess for this age group. Both personal income and household income have even lower relevance to older youth and young adults: typically, young adults from the higher socioeconomic backgrounds are in university and often report to have the lowest or no income during this period. Perceived income adequacy is an alternative measure that assesses the level of need, which has a more consistent meaning across young adults in different educational and occupational settings. Weight status and self-reported general health were useful predictors for food purchasing behaviors in previous studies [40], and are thus included in the present study. Weight status in this study was derived from Body Mass Index (BMI) calculated by self-reported height and weight, the validity of which was recognized by previous studies [41]. Additionally, responses to a question about weight concern (how strong a participant agreed with the statement “I worry about becoming fat”) are included to control for personal attitudes that may affect dietary behaviours. The likelihood ratio test shows that the interaction term of weight concerns and sex is not a significant predictor of the number of fast food meals consumed, and the inclusion of this variable has a small effect on the coefficients and confidence intervals of other selected predicting variables, except sex. Therefore, the final models do not include a variable accounting for the interaction between worrying about weights and sex. Distributions of these covariates are shown in Table 2.

**Table 2:** Distributions of age, sex, income adequacy, weight status, general health, weight concern, city of residence, and survey wave

Variables	Respondents	Variables	Respondents
**Age**		**Weight status**	
16–18	101 (17.1%)	Underweight (BMI < 18.5)	45 (7.6%)
19–21	188 (31.8%)	Normal weight (18.5 ≤ BMI < 24.9)	371 (62.8%)
22–25	166 (28.1%)	Overweight (24.9 ≤ BMI < 29.9)	121 (20.5%)
26–30	136 (23.0%)	Obese (BMI ≥ 29.9)	54 (9.1%)
**Sex**		**General health**	
Female	384 (65.0%)	Poor	24 (4.1%)
Male	207 (35.0%)	Fair	151 (25.5%)
**Income adequacy** (How difficult or easy is it to make ends meet?)		Good	257 (43.5%)
Very difficult	46 (7.8%)	Very good	143 (24.2%)
Difficult	119 (20.1%)	Excellent	16 (2.7%)
Neither easy nor difficult	251 (42.5%)	**Weight concern** (I’m worrying about becoming fat.)	
Easy	126 (21.3%)	Strongly disagree	67 (11.3%)
Very easy	49 (8.3%)	Disagree	67 (11.3%)
**Residential city**		Neutral	125 (21.2%)
Toronto	166 (28.1%)	Agree	204 (34.5%)
Montreal	78 (13.2%)	Strongly agree	128 (21.7%)
Halifax	131 (22.2%)	**Wave**	
Edmonton	90 (15.2%)	2016	356 (60.2%)
Vancouver	126 (21.3%)	2017	235 (39.8%)

### Negative binomial regression

Negative Binomial Regression (NBR) was chosen as our vehicle for modelling associations between fast food exposure and consumption because of the over-dispersed distribution of the count of fast food consumption. This analysis was conducted using stats v3.6.1 package for R [42].

### Sensitivity analyses

To test the sensitivity of our results to changes in how the activity space-based exposure variables were calculated, buffers of 500 m, 1 km, and 1.5 km were used to generate activity spaces. Results from models using all three distances are presented in the following section.

## Results

**Fig. 3:**
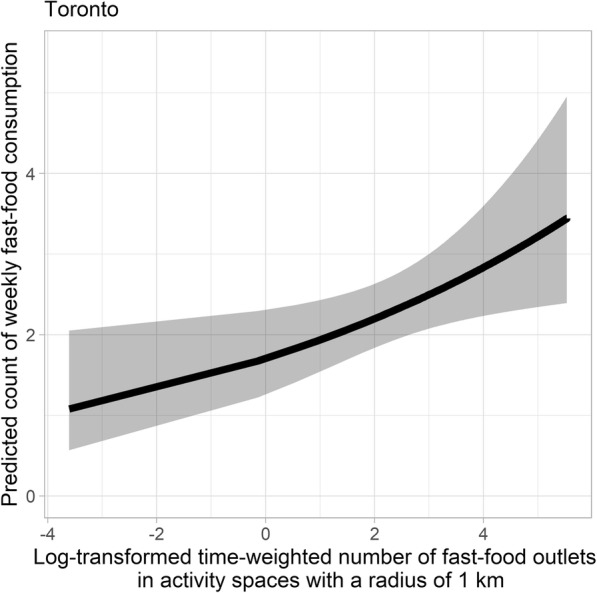
Predicted count of fast food meals against log-transformed time-weighted number of fast food outlets for young adults residing in Toronto. Note: The ribbons illustrate 95% confidence intervals. Results shown in this figure are derived from the model when 1 km buffers are used. The range of log-transformed time-weighted number of fast food outlets for respondents in five cities is (− 3.609, 5.531), corresponding to the range of time-weighted number of fast food outlets (0.027, 252.28). Age, sex, income adequacy, weight status, general health status, weight concern, ALE measure, and city of residence are adjusted for

**Fig. 4:**
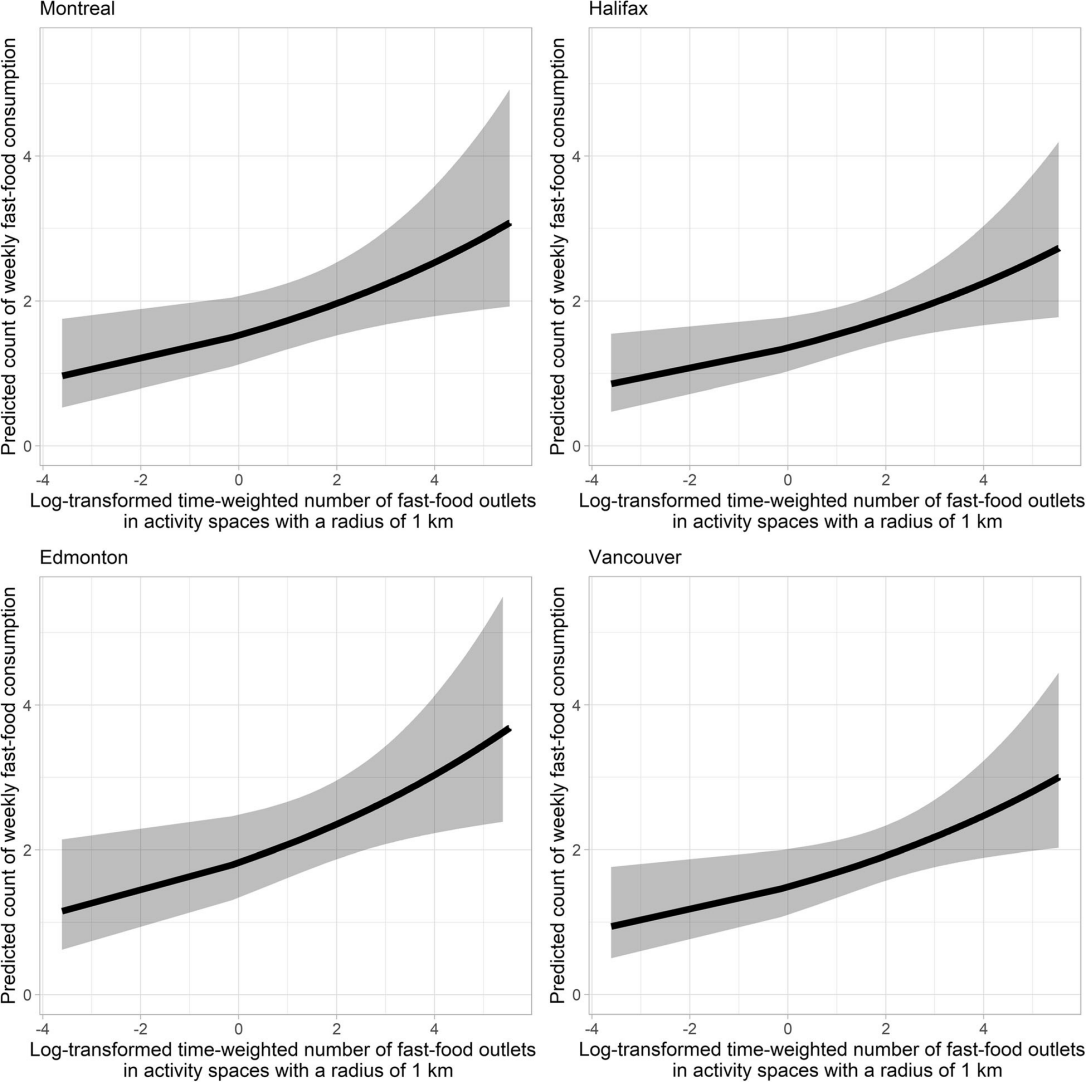
Predicted count of fast food meals against log-transformed time-weighted number of fast food outlets for young adults residing in Montreal (top left), Halifax (top right), Edmonton (bottom left), and Vancouver. Note: The ribbons illustrate 95% confidence intervals. Results shown in this figure are derived from the model when 1 km buffers are used. Age, sex, income adequacy, weight status, general health status, weight concern, ALE measure, and city of residence are adjusted for

Significant positive associations were found between the log-transformed time-weighted number of fast food outlets present within activity spaces, and the weekly frequency of fast food consumption, using all three exposure buffer distances (Table 3). This positive relationship was also consistent across the five study cities (Figs. 3 and 4). For one-unit increase in the log-transformed time-weighted fast food outlet number, the expected weekly count of fast food consumption increased by 7.8% (95% CI: − 0.1, 16.3%), 13.5% (95% CI: 2.4, 25.9%), and 13.8% (95% CI: 0.4, 28.9%) when a radius of 500 m, 1 km, and 1.5 km was used, respectively (Table 3; See Additional file 3 for detailed model output of estimates of all predictor variables and model fit statistics). To put this into context, for a subsample of young adults residing in Toronto, weekly count of fast food meals is expected to increase from 2.093 (95% CI: 1.726, 2.538) to 2.406 (95% CI: 2.016, 2.872) with an increase from 5 to 15 in the time-weighted number of fast food outlets in their 1 km activity spaces, holding covariates constant.

**Table 3:** Associations of time-weighted count of fast food outlets in participants’ activity spaces with count of fast food consumption estimated using a negative binomial regression model among young adults (*n* = 591) in five Canadian urban regions

	Model 1^a^		Model 2		Model 3	
	IRR ^b^	95% CI	IRR	95% CI	IRR	95% CI
Log transformed time-weighted number of fast food stores ^c^	1.078*	0.999, 1.163	1.135**	1.024, 1.259	1.138**	1.004, 1.289

*P < 0.1, **P < 0.05, ***P < 0.01

^a^ Models 1, 2, 3 adjust for age, sex, income adequacy, weight status, general health status, weight concern, ALE measure, and city of residence. Log transformed time-weighted number of fast food stores are computed in buffer of activity locations with radii of 500 m, 1 km, and 1.5 km in Models 1, 2, 3 respectively

^b^ Incident rate ratios (IRRs) represent difference in expected weekly fast food consumption frequency per one-unit increase in a predictor variable

^c^ The temporal weight is the proportion of time spent in each of the activity locations relative to the total time spent in all activity locations of an individual

**Fig. 5:**
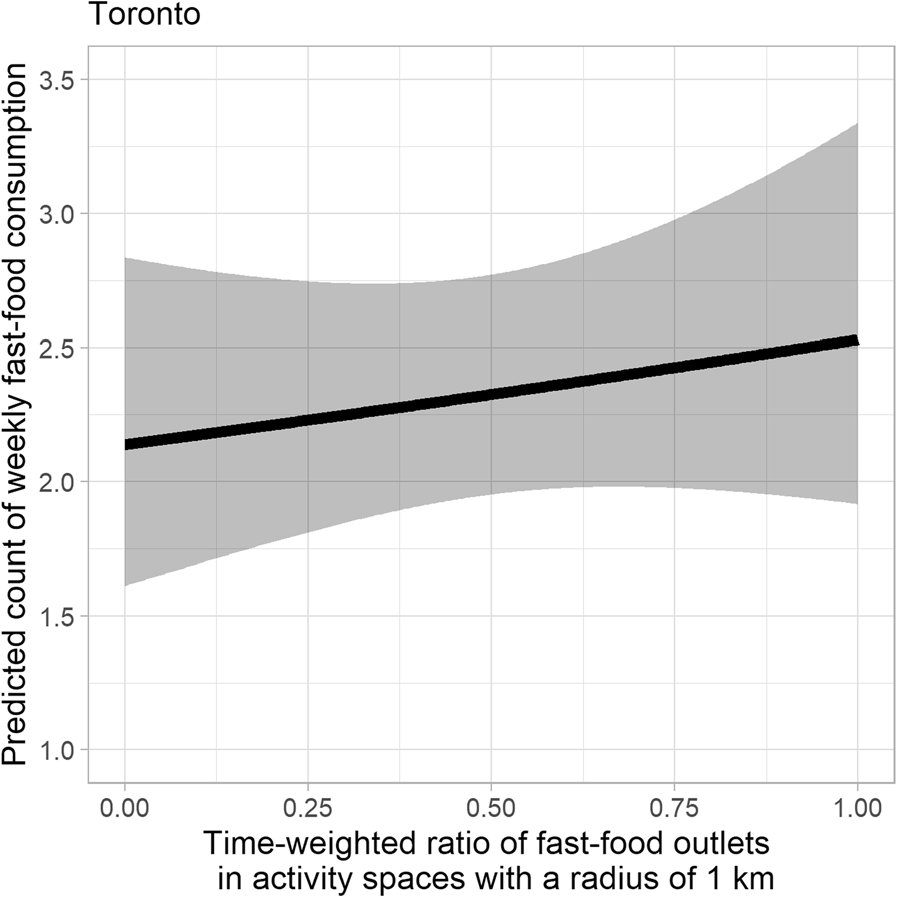
Predicted count of fast food meals against time-weighted ratio of fast food outlets for young adults residing in Toronto. Note: The ribbons illustrate 95% confidence intervals. Results shown in this figure are derived from the model when 1 km buffers are used. Age, sex, income adequacy, weight status, general health status, weight concern, ALE measure, and city of residence are adjusted for

**Fig. 6:**
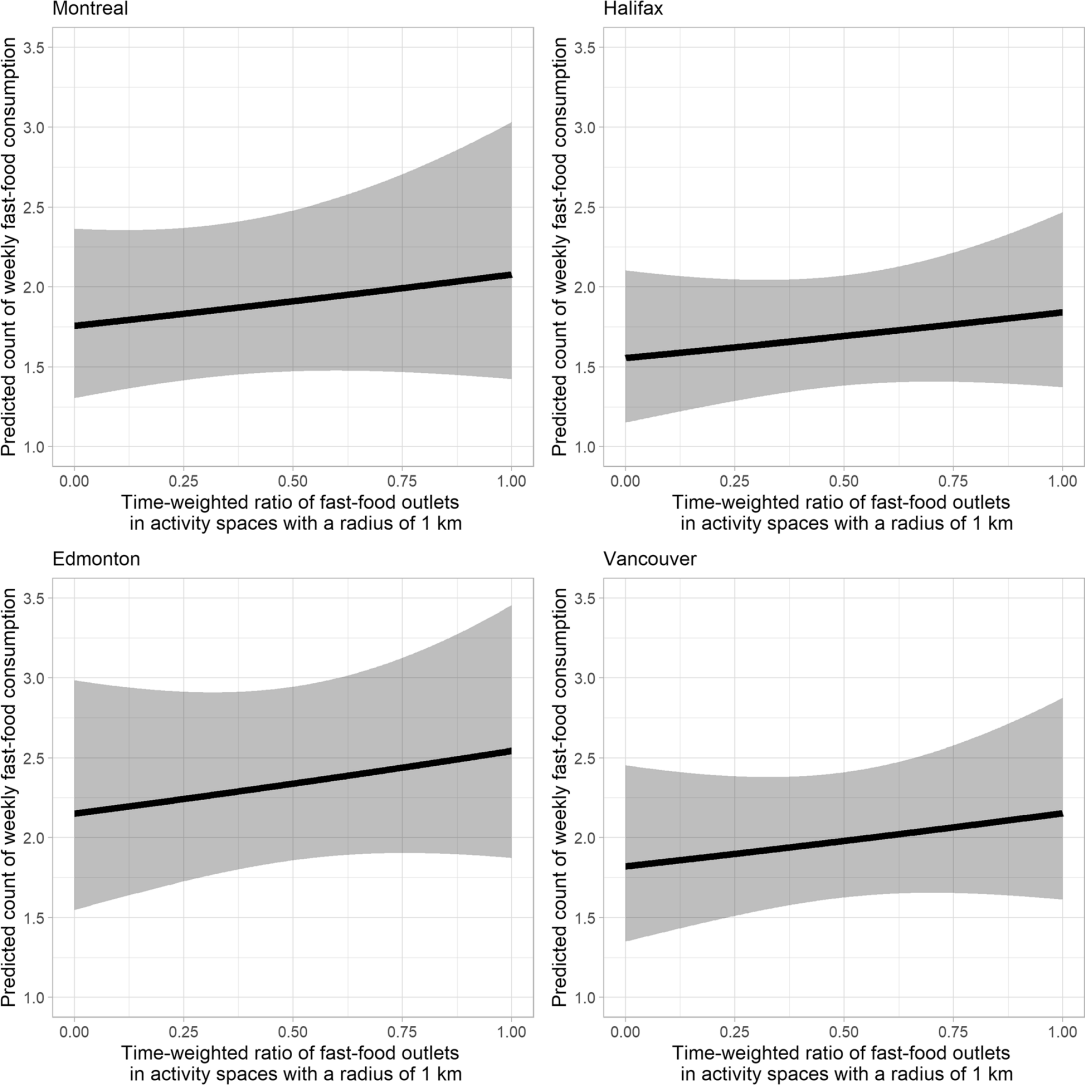
Predicted count of fast food meals against time-weighted ratio of fast food outlets for young adults residing in Montreal (top left), Halifax (top right), Edmonton (bottom left), and Vancouver. Note: The ribbons illustrate 95% confidence intervals. Results shown in this figure are derived from the model when 1 km buffers are used. Age, sex, income adequacy, weight status, general health status, weight concern, ALE measure, and city of residence are adjusted for

Regarding the relative exposure measure, significant associations are shown in the models that employ a radius of 500 m, with a 10 percentage point greater time-weighted ratio of fast food outlets corresponding to a 4.78% (95% CI: 0.32, 11.23%) greater expected count of weekly fast food consumption (Table 4). Again, to put this into context, for the sample of young adults from Toronto, the counts of fast food meals are expected to be 2.183 (95% CI: 1.814, 2.627) and 2.654 (95% CI: 2.154, 3.270), respectively, with 0.25 and 0.75 as the time-weighted ratios of fast food outlets in 500 m activity spaces. Thus, an increase in the time-weighted ratio of fast food outlets from 0.25 to 0.75 is associated with an increase of approximately 0.5 additional fast food meals in a week. However, this relative measure of exposure is not a significant predictor when either a radius of 1 km or 1.5 km is used. Figures 5 and 6 show the insignificant positive trend when the 1 km buffer is used across five cities.

**Table 4:** Associations of time-weighted ratio of fast food outlets in participants’ activity spaces with count of fast food consumption estimated using a negative binomial regression model among young adults (*n* = 591) in five Canadian urban regions

	Model 4^a^		Model 5		Model 6	
	IRR ^b^	95% CI	IRR	95% CI	IRR	95% CI
Time-weighted ratio of fast food stores ^c^	1.478**	1.032, 2.123	1.183	0.759, 1.847	1.263	0.730, 2.182

*P < 0.1, **P < 0.05, ***P < 0.01

^a^ Models 4, 5, 6 adjust for age, sex, income adequacy, weight status, general health status, weight concern, ALE measure, and city of residence. Time-weighted ratio of fast food stores are computed in buffer of activity locations with radii of 500 m, 1 km, and 1.5 km in Models 4, 5, 6 respectively

^b^ Incident rate ratios (IRRs) represent difference in expected weekly fast food consumption frequency per one-unit increase in a predictor variable

^c^ The temporal weight is the proportion of time spent in each of the activity locations relative to the total time spent in all activity locations of an individual

Finally, the two variables describing the urban context of participants produced significant relationships. First, the time-weighted Active Living Environment Index with Transit measure that characterizes overall built environment is negatively related to the count of fast food meals in models using the absolute or relative measures of fast- food exposure, across all buffer thresholds (see Additional file 3). This implies that the likelihood of young adults consuming fast food meals more frequently is lower in denser urban environments. In addition to the built environment variable, the urban dummy variables demonstrated regional differences in the count of fast food meals across the study population. Participants residing in Halifax had significantly fewer fast food meals compared with Toronto, while the counts of fast food consumption by respondents from Montreal, Edmonton, and Vancouver did not significantly deviate from Toronto (see Additional file 3).

## Discussion

In this analysis of 591 young adults from five Canadian cities, we found consistent associations between absolute time-weighted activity space-based fast food exposure and the frequency of participants’ fast food meal consumption in models using activity space buffers with radii of 500 m, 1 km, and 1.5 km. Time-weighted proportion of fast food outlets, the relative measure of fast food exposure, was found to be significantly associated with weekly fast food consumption frequency when a 500 m buffer is used around activity locations. However, significant associations were not found when the relative measure of fast food exposure was derived from activity space buffers with radii of 1 km and 1.5 km. In addition, this study found that the time-weighted Canadian Active Living Environment Index with Transit measures were significantly and negatively associated with the weekly count of fast food meals of young, urban Canadians, indicating young adults who were active in urban environment with denser built infrastructure and amenities tended to consume fast food meals less frequently.

### Associations between exposure to fast food outlets and counts of fast food consumption among young adults

The positive associations found between fast food exposure and the count of fast food restaurant meals were consistent with those of recent research, including studies using absolute measures of fast food exposure [12, 23, 36] and those using relative measures [24, 43]. However, the positive associations using time-weighted absolute exposure at distances up to 1.5 km shown in this study vary from a previous study that used time-weighted counts of fast food restaurant exposure that only showed positive associations with fast food restaurant visits at 21 m and 100 m proximities, but not at distances of 500 m and 0.5mile [23]. The disparity may be due to different study populations (young adults aged 16–30 in this study, versus primary household food shoppers aged 18–65), study regions, characterization of outcome variable (count of fast food meals consumed in this study versus one or more fast food restaurant visits), and the methods of defining activity spaces from GPS data (identifying activity locations from GPS points where at least 10 min was spent in this study versus intersecting line segments derived from each pair of GPS points with the areas within fast food restaurant proximity buffers). More evidence is required to determine whether absolute or relative exposure measures are appropriate for various types of food retailers, and in what circumstances different distance thresholds should be used.

In this study, significant positive associations were found for all activity space sizes considered for absolute fast food outlet exposure, while a significant association was only observed with a 500 m radius in models using a relative measure of fast food outlet exposure. Given the predictions noted in the results section for a subsample of young adults residing in Toronto, an increase from 5 to 15 in the time-weighted number of fast food outlets measured using 1 km activity spaces corresponds to the consumption of approximately 16 additional fast food meals over a 52 week period. Similarly, an increase from 0.25 to 0.75 in the time-weighted ratios of fast food outlets in 500 m activity spaces is associated with approximately 24 additional fast food meals over a 52-week period.

### Strengths and limitations

Use of activity space approaches goes beyond residential neighborhoods and more accurately captures individuals’ exposure to retail food environments in the course of temporally and spatially defined life activities [19, 44]. Moreover, it is possible to account for the duration of exposure to the food environments a person experiences throughout some period, which has rarely been considered when an activity space approach is used. Given the assumption that foodscapes around places where a person spends more time are more representative of overall food environment exposures s/he experiences than places where less time is spent, higher weights are assigned for the former when computing the exposure measure. These two major methodological advantages of time-weighted exposure measures employed in this study may improve the validity of analyses on the link between retail food environment exposure and fast food consumption.

However, the study is not without its limitations. The sample size of this study, which is commensurate with or a little larger than other recent studies using GPS technologies [6, 23], is still relatively small, which may limit the generalizability of the findings. Due to the feasibility of implementation, the GPS data were collected 1 month after the questionnaire in which the weekly counts of fast food meals were reported. We would suggest, however, that contemporaneous data collection would not have meaningfully changed the results, given the habitual nature of mobility patterns in humans [45]. Errors in the geographical coordinates produced by GPS tracking technology and a lack of supplementary information on participants’ visits to fast food outlets prevent us from deriving where and when fast food outlets visits were made. Thus, the implications for a causal link between exposure and consumption may be affected by the selective mobility bias, which describes the phenomenon where exposure to fast food outlets is biased when an individual intentionally visits a fast food retailer to purchase a meal [46]. This problem could be solved in future research by excluding activity spaces where the central activity was the act of consuming fast food [46, 47]. Moreover, timing or time of the day, an important factor associated with food consumption behaviours, has not been accounted for in this study because of the limited capability to identify the use of fast food outlets using our data. Future studies should explore whether exposures at various times of the day affect fast food consumption behaviours. Another dimension of food consumption behaviour that has not been addressed is that food intake is socially-mediated, particularly for younger adults. These social influences may also have geographic components that influence food choices. However, this study does not have any information on participants’ social networks, limiting the ability to conduct such an analysis. In addition, this study is limited by a lack of officially validated food retail data, because no disaggregated official business register of food retailers was accessible to the public in Canada at the time of this research. It may lead to measurement errors in exposure measures and thus may bring about differences in the size of the associations between food environment and weight status [48, 49]. However, previous studies have used OpenStreetMap as an source of food outlet data [50, 51], and numbers of fast food outlets at the DA level from OpenStreetMap and an officially validated dataset [52] were highly correlated in the five Canadian urban regions.

## Conclusions

This study related characteristics of activity spaces to frequency of fast food meal consumption among young adults from five Canadian cities. Our results demonstrated that absolute activity space-based measures of fast food exposure using buffer radii of 500 m, 1 km, and 1.5 km, and relative activity space-based measures using a 500 m buffer, were significantly positively associated with frequency of fast food consumption. This novel activity space-based approach not only accounts for individuals’ movement patterns, but also considers the duration of exposure, which to date has been under-researched. Beyond this, this study found that the time-weighted Canadian Active Living Environment Index with Transit measures were negatively associated with the weekly count of fast food meals of young, urban Canadians, suggesting that participants who were more active in dense urban environments tended to consume fast food meals less frequently. Ultimately, this paper strengthens the case for using activity space-based measures to understand how the food environment affects diets, and also makes a new case that more nuanced accountings of the urban built environment, beyond describing food retail, can improve models focused on how urban form can affect dietary behaviours.

## Supplementary information

**Additional file 1.** Information about sample. Information regarding how the sample was recruited; how representative the sample was of the target group; how the analysed sample differed from the recruited sample; and how any missing data were handled.

**Additional file 2.** Dissemination area (DA) and census metropolitan area (CMA) in Canada. Information regarding two types of administrative geographical units mentioned in the analysis.

**Additional file 3.** Sensitivity analyses using buffers with radii of 500 m, 1 km, and 1.5 km. Model results and model fit statistics for sensitivity analyses using buffers with radii of 500 m, 1 km, and 1.5 km.

## Data Availability

The datasets analysed during the current study are not publicly available due to protection of respondents’ privacy but are available from the corresponding author on reasonable request.

## Abbreviations

ALE_TRANSIT: Active Living Environment Index with transit z score included
BMI: Body Mass Index
Can-ALE: Canadian Active Living Environments database
CFS: Canada Food Study
CMA: Census metropolitan area
DA: Dissemination area
GPS: Global Positioning System
IRR: Incident rate ratio
KDE: Kernel density estimate
NAICS: North American Industry Classification System
NBR: Negative binomial regression
OSM: OpenStreetMap
UGCoP: Uncertain Geographic Context Problem
