# Supplementary material for: Association between time-weighted activity space-based exposures to fast food outlets and fast food consumption among young adults in urban Canada
Source: Int J Behav Nutr Phys Act. 2020 May 13;17:62. doi: 10.1186/s12966-020-00967-y (PMC7222540; doi:10.1186/s12966-020-00967-y)
Supplement: Supplementary file 1 — Additional file 1. Information about sample. Information regarding how the sample was recruited; how representative the sample was of the target group; how the analysed sample differed from the recruited sample; and how any missing data were handled. [file 12966_2020_967_MOESM1_ESM.docx]

**Additional file 1 Information about sample**

### How the sample was recruited

Data come from the Canada Food Study (CFS). Respondents were recruited from five Canadian cities: Vancouver, Edmonton, Toronto, Montreal, and Halifax. Individuals were eligible to participate if they resided in one of the five cities, were 16-30 years old at the time of recruitment, had access to the internet, as well as a laptop, desktop computer or tablet [1].

Participants were recruited using in-person intercept recruitment from selected sites in each city, using a stratified sample of sites. For each city, a sampling frame of shopping centres and public areas was constructed, stratified by city region/neighbourhood and type of site (mall, transit hub, park, or other shopping district). Each city was stratified into 3-5 regions based on neighbourhood boundaries. In each region, two sites were selected within each of the four site type strata where feasible [1].

Through this process of recruitment, 6,720 respondents were recruited in Wave 2016. Of these, 3,234 accessed the survey link for a cooperation rate of 48.1%. As a data integrity check, respondents were asked midway through the main survey to select the current month from a list. The month selected by the respondent was compared to the month when the survey was submitted. Respondents with month discrepancies were excluded from the analytic sample. Overall, 41 respondents were excluded from the analytic sample due to discrepancies with the month selected. Two additional respondents were excluded from the analytic sample due to other data quality concerns (i.e., unusual/suspicious responses for several questions). The final main survey sample in Wave 2016 included 3,000 respondents [1].

All respondents in the analytic sample in Wave 2016 were sent an email invitation for Wave 2017 of the CFS, with the exception of eight respondents (n=2,992). 1,115 accessed the survey link, for a re-contact rate of 37.3%. Of those, 51 were disqualified for ineligibility and 42 were excluded due to data quality concerns (using similar data integrity check employed in Wave 2016). The final sample size in Wave 2017 is 1,022 with a follow-up rate of 34.1% [2].

1. **How representative the sample was of the target group**

To show that the sample recruited in CFS Wave 2016 was representative of young adults residing in Canadian urban regions, we compared CFS Wave 2016 sample with young adults cohort from the 2014 Canadian Community Health Survey (CCHS), a large sample nationwide health survey [3]. Briefly, the CFS study sample was more highly educated (more than high school, CFS=91% vs. CCHS=70%), and more likely to be currently be a student (CFS=52% vs. CCHS=29%). CFS and CCHS respondents reported very similar levels of overweight and obesity (CFS=32% vs. CCHS=36%) and perceived weight status (CFS=26% “overweight” vs. CCHS=26%) [1].

Table 1. Sample comparison between the Canada Food Study (CFS) Wave 2016 and the 2014 Canadian Community Health Survey (CCHS).

| **Measure** | **Canada Food Study (CFS)** | **National Estimates in 2014 Canadian Community Health Survey (CCHS)** |
| --- | --- | --- |
| Highest level of education | 2016 CFS, 20-29 year olds  Education Level (S1A_educ_level_DV)  High school or less: 8.8%  CEGEP/Trade/college/university (partial or complete): 91.2% | 2014 CCHS, 20-29 year olds  Highest level of education – respondent 4 levels (EDUDR04)  Sec. School or less: 30.2%  Post-Sec Ed (partial or complete): 69.8% |
| Current student | 2016 CFS, 20-29 year olds  Are you currently a student? (S1A_student)  Yes: 52.2%  No: 47.8% | 2014 CCHS, 20-29 year olds  Are you currently attending a school, college or university? (SDC_8)  Yes: 29.0%  No: 71.0% |
| BMI Category | 2016 CFS, 18-29 year olds  BMI Class (S1A_BMI_class_DV)  Underweight: 6.6%  Normal weight: 61.8%  Overweight: 21.8%  Obese: 9.7%  Percent with Missing:  Underweight: 5.5%  Normal weight: 51.3%  Overweight: 18.1%  Obese: 8.1%  Missing: 17.0% | 2014 CCHS, 18-29 year olds  BMI Class (HWTGISW)  Underweight: 4.9%  Normal weight: 59.5%  Overweight: 22.8%  Obese: 12.8%  Percent with Not Stated:  Underweight: 4.6%  Normal weight: 55.8%  Overweight: 21.4%  Obese: 12.0%  Not stated: 6.2% |
| Perceived weight status | 2016 CFS, 18-29 year olds  Do you consider yourself… (S1A_wt_perceive)  Overweight: 25.6%  Underweight: 9.7%  Just about right: 64.7 % | 2014 CCHS, 18-29 year olds  Do you consider yourself… (HWT_4)  Overweight: 26.2%  Underweight: 6.9%  Just about right: 66.9% |

1. **How the analysed sample differed from the recruited sample**

A sub-sample of CFS cohort members were recruited for the GPS Survey. GPS Survey was collected over seven consecutive days for a subsample of 630 respondents in Wave 2016 and 400 in Wave 2017 using a version of the Itinerum smartphone application [4]. For this analysis, respondents who participated in the GPS Survey for less than 72 hours, as judged by the length of time the smartphone application was installed, were excluded, resulting in a study sample of 575 participants in 2016 and 373 in 2017. For those respondents who participated in both waves of GPS data collection, the wave with the longer recorded time period, in hours, of GPS data was retained, which resulted in a sample of 728 participants. A further 137 individuals were excluded due to missing data in other relevant variables. The final analytical sample included 591 participants, of which, 356 were from Wave 2016 and 235 from Wave 2017.

The analysed sample has a high representativeness of the recruited sample in terms of the distributions of age groups, sex, residential city, and weight status (Table 2), except that there are fewer respondents in the 16 – 18 age group or from Montreal in the analysed sample.

Table 2. Comparison of analysed sample (n = 591) and recruited sample (n = 3,000)

| **Variables** | **Analysed sample (n = 591)** | **Recruited sample (n = 3,000)** |
| --- | --- | --- |
| **Age** |  |  |
| 16 - 18 | 17.1% (101) | 24.4% (731) |
| 19 - 21 | 31.8% (188) | 29.2% (876) |
| 22 - 24 | 21.0% (124) | 21.9% (656) |
| 25 - 27 | 19.6% (116) | 14.7% (442) |
| 28 - 30 | 10.5% (62) | 9.8% (295) |
| **Sex** |  |  |
| Female | 65.0% (384) | 60.5% (1816) |
| Male | 35.0% (207) | 39.5% (1184) |
| **Residential city** |  |  |
| Toronto | 28.1% (166) | 25.5% (765) |
| Montreal | 13.2% (78) | 18.7% (562) |
| Halifax | 22.2% (131) | 19.4% (582) |
| Edmonton | 15.2% (90) | 17.2% (516) |
| Vancouver | 21.3% (126) | 19.2% (575) |
| **Weight status** |  |  |
| Underweight | 7.6% (45) | 6.9% (206) |
| Normal weight | 62.8% (371) | 50.8% (1524) |
| Overweight | 20.5% (121) | 15.7% (471) |
| Obese | 9.1% (54) | 7.8% (235) |
| Not stated | 0.0% (0) | 18.8% (564) |

1. **How any missing data were handled**

Data entries of 137 individuals (out of 728 respondents with valid GPS data) with missing values in the response variable (count of weekly fast-food meals consumed) and/or covariates (age, sex, income adequacy, residential city, weight status, general health, weight concern) are omitted. Thus, there are no missing data in the analysed sample.

**References**

1. Hammond D, White CM, Reid JL. Canada Food Study: Technical Report – Wave 1 (2016) [Internet]. 2019 [cited 2019 Feb 20]. Available from: http://canadafoodstudy.ca/studydocs

2. Hammond D, White CM, Reid JL. Canada Food Study: Techinical report – Wave 2 (2017). [Internet]. 2019 [cited 2019 Feb 20]. Available from: http://canadafoodstudy.ca/studydocs

3. Statistics Canada. Canadian Community Health Survey – Annual Component: Detailed Information for 2014 [Internet]. 2015. Available from: http://www23.statcan.gc.ca/imdb/p2SV.pl?Function=getSurvey&Id=164081

4. Patterson, Z., Fitzsimmons, K., Jackson, S., & Mukai T. Itinerum: The open smartphone travel survey platform. SoftwareX. 2019;10:100230.
