# Supplementary material for: Association between time-weighted activity space-based exposures to fast food outlets and fast food consumption among young adults in urban Canada
Source: Int J Behav Nutr Phys Act. 2020 May 13;17:62. doi: 10.1186/s12966-020-00967-y (PMC7222540; doi:10.1186/s12966-020-00967-y)
Supplement: Supplementary file 2 — Additional file 2. Dissemination area (DA) and census metropolitan area (CMA) in Canada. Information regarding two types of administrative geographical units mentioned in the analysis. [file 12966_2020_967_MOESM2_ESM.docx]

**Additional file 2 Dissemination area (DA) and census metropolitan area (CMA) in Canada**

1. **Dissemination area (DA)**

Dissemination area (DA) is defined by Statistics Canada [1] as “small area composed of one or more neighbouring dissemination blocks, with a population of 400 to 700 persons.” The average area sizes of DAs in Census Metropolitan Areas of Toronto, Montreal, Halifax, Edmonton, and Vancouver are 0.872, 0.738, 10.284, 5.666, and 0.879 square kilometers, which were calculated by the authors using ArcMap 10.6 (Esri Inc., Redlands, USA; http://desktop.arcgis.com/en/arcmap/).

1. **Census metropolitan area (CMA)**

Census metropolitan area (CMA) is defined by Statistics Canada [2] as “area consisting of one or more neighbouring municipalities situated around a core”, and a CMA “must have a total population of at least 100,000 of which 50,000 or more live in the core”.

**References**

1. Statistics Canada. Dissemination area (DA) [Internet]. 2015 [cited 2018 Nov 30]. Available from: https://www12.statcan.gc.ca/census-recensement/2011/ref/dict/geo021-eng.cfm

2. Statistics Canada. Census metropolitan area (CMA) and census agglomeration (CA) [Internet]. 2015 [cited 2018 Nov 30]. Available from: https://www12.statcan.gc.ca/census-recensement/2011/ref/dict/geo009-eng.cfm
