# Supplementary material for: Association between time-weighted activity space-based exposures to fast food outlets and fast food consumption among young adults in urban Canada
Source: Int J Behav Nutr Phys Act. 2020 May 13;17:62. doi: 10.1186/s12966-020-00967-y (PMC7222540; doi:10.1186/s12966-020-00967-y)
Supplement: Supplementary file 3 — Additional file 3. Sensitivity analyses using buffers with radii of 500 m, 1 km, and 1.5 km. Model results and model fit statistics for sensitivity analyses using buffers with radii of 500 m, 1 km, and 1.5 km. [file 12966_2020_967_MOESM3_ESM.docx]

**Additional file 3 Sensitivity analyses using buffers with radii of 500 m, 1 km, and 1.5 km**

1. **Models exploring how the time-weighted count of fast food outlets in participants’ activity spaces relates to count of weekly fast food consumption employing buffers with a radius of 500 m, 1 km, and 1.5 km**

Table 1. Models exploring how the time-weighted count of fast food outlets in participants’ activity spaces relates to count of weekly fast food consumption.

|  | Count of weekly fast-food consumption | | | | | | | | |
| --- | --- | --- | --- | --- | --- | --- | --- | --- | --- |
|  | Using a 500m buffer | | | Using a 1000m buffer | | | Using a 1500m buffer | | |
|  | IRR | 2.5 % | 97.5 % | IRR | 2.5 % | 97.5 % | IRR | 2.5 % | 97.5 % |
| Intercept | 5.336*** | 2.488 | 11.497 | 4.409*** | 2.035 | 9.590 | 4.018*** | 1.800 | 9.006 |
| Age | 1.020 | 0.995 | 1.046 | 1.020 | 0.995 | 1.046 | 1.019 | 0.994 | 1.046 |
| Sex (Ref = female) | 1.183* | 0.971 | 1.441 | 1.172 | 0.964 | 1.427 | 1.173 | 0.965 | 1.429 |
| Income adequacy | 0.929 | 0.848 | 1.017 | 0.928 | 0.848 | 1.016 | 0.929 | 0.849 | 1.018 |
| BMI | 0.967 | 0.849 | 1.101 | 0.968 | 0.850 | 1.101 | 0.971 | 0.854 | 1.106 |
| General health | 0.792*** | 0.711 | 0.881 | 0.796*** | 0.715 | 0.886 | 0.793*** | 0.712 | 0.882 |
| Weight bias | 0.912** | 0.846 | 0.981 | 0.915** | 0.849 | 0.984 | 0.917** | 0.852 | 0.987 |
| Log transformed time-weighted number of fast food outlets | 1.078* | 0.999 | 1.163 | 1.135** | 1.024 | 1.259 | 1.138** | 1.004 | 1.289 |
| Montreal (Ref = Toronto) | 0.873 | 0.638 | 1.196 | 0.894 | 0.653 | 1.225 | 0.888 | 0.647 | 1.221 |
| Halifax (Ref = Toronto) | 0.777* | 0.587 | 1.028 | 0.793 | 0.599 | 1.050 | 0.783* | 0.591 | 1.036 |
| Edmonton (Ref = Toronto) | 1.061 | 0.785 | 1.435 | 1.070 | 0.793 | 1.446 | 1.091 | 0.804 | 1.482 |
| Vancouver (Ref = Toronto) | 0.868 | 0.666 | 1.132 | 0.871 | 0.669 | 1.135 | 0.880 | 0.674 | 1.148 |
| Time-weighted CanALE index with transit measure | 0.975*** | 0.957 | 0.994 | 0.967*** | 0.946 | 0.989 | 0.968*** | 0.945 | 0.991 |
| Observations | 591 |  |  | 591 |  |  | 591 |  |  |
| 2 Log likelihood | -2270.768 |  |  | -2268.663 |  |  | -2270.304 |  |  |
| θ | 1.384 |  |  | 1.395 |  |  | 1.387 |  |  |
| θ_SE_ | 0.149 |  |  | 0.151 |  |  | 0.149 |  |  |
| AIC | 2298.8 |  |  | 2296.7 |  |  | 2298.3 |  |  |

Participants were young urban adults (n = 591) aged 16–30 years, a subset of respondents in the 2016 Canada Food Study (CFS).

Beta coefficients are presented as incident rate ratios (IRRs), with 90% confidence intervals (CIs).

AIC, Akaike information criterion.

The reference category of sex is female.

The reference category of cities is Toronto.

The time-weighted density of built environment is the time-weighted sum of ALE_TRANSIT (Active Living Environment Index with transit z score included) in the dissemination areas (DAs) containing the activity locations of an individual. Derived from Canadian Active Living Environments Database (Can-ALE) 2016, ALE_TRANSIT is the sum of the z-score of the intersection density, dwelling density, points of interest, and transit measures [1].

The temporal weight is the proportion of time spent in each of the activity locations relative to the total time spent in all activity locations of an individual.

*P<0.1, **P<0.05, ***P<0.01.

1. **Models exploring how the time-weighted ratio of fast food outlets in participants’ activity spaces relates to count of weekly fast food consumption employing buffers with a radius of 500 m, 1 km, and 1.5 km**

Table 2. Models exploring how the time-weighted ratio of fast food outlets in participants’ activity spaces relates to count of weekly fast food consumption.

|  | Count of weekly fast-food consumption | | | | | | | | |
| --- | --- | --- | --- | --- | --- | --- | --- | --- | --- |
|  | Using a 500m buffer | | | Using a 1000m buffer | | | Using a 1500m buffer | | |
|  | IRR | 2.5 % | 97.5 % | IRR | 2.5 % | 97.5 % | IRR | 2.5 % | 97.5 % |
| Intercept | 4.663*** | 2.163 | 10.097 | 4.771*** | 2.138 | 10.700 | 4.636*** | 2.051 | 10.530 |
| Age | 1.022* | 0.996 | 1.048 | 1.021 | 0.995 | 1.047 | 1.021 | 0.995 | 1.047 |
| Sex (Ref = female) | 1.163 | 0.956 | 1.415 | 1.158 | 0.952 | 1.411 | 1.154 | 0.948 | 1.405 |
| Income adequacy | 0.933 | 0.852 | 1.022 | 0.934 | 0.853 | 1.023 | 0.933 | 0.852 | 1.022 |
| BMI | 0.966 | 0.849 | 1.100 | 0.977 | 0.858 | 1.112 | 0.979 | 0.860 | 1.115 |
| General health | 0.790*** | 0.709 | 0.878 | 0.796*** | 0.715 | 0.885 | 0.794*** | 0.713 | 0.884 |
| Weight bias | 0.918** | 0.853 | 0.988 | 0.918** | 0.852 | 0.988 | 0.916** | 0.850 | 0.986 |
| Time-weighted ratio of fast food outlets | 1.478** | 1.032 | 2.123 | 1.183 | 0.759 | 1.847 | 1.263 | 0.730 | 2.182 |
| Montreal (Ref = Toronto) | 0.850 | 0.626 | 1.156 | 0.821 | 0.604 | 1.119 | 0.826 | 0.606 | 1.127 |
| Halifax (Ref = Toronto) | 0.724** | 0.551 | 0.951 | 0.728** | 0.553 | 0.956 | 0.719** | 0.545 | 0.947 |
| Edmonton (Ref = Toronto) | 1.014 | 0.754 | 1.366 | 1.005 | 0.747 | 1.356 | 1.004 | 0.745 | 1.353 |
| Vancouver (Ref = Toronto) | 0.856 | 0.658 | 1.116 | 0.851 | 0.653 | 1.109 | 0.851 | 0.653 | 1.109 |
| Time-weighted CanALE index with transit measure | 0.979** | 0.963 | 0.996 | 0.984** | 0.968 | 1.000 | 0.984** | 0.968 | 1.000 |
| Observations | 591 |  |  | 591 |  |  | 591 |  |  |
| 2 Log likelihood | -2269.862 |  |  | -2273.846 |  |  | -2273.696 |  |  |
| θ | 1.389 |  |  | 1.370 |  |  | 1.370 |  |  |
| θ_SE_ | 0.149 |  |  | 0.147 |  |  | 0.147 |  |  |
| AIC | 2297.9 |  |  | 2301.8 |  |  | 2301.7 |  |  |

Participants were young urban adults (n = 591) aged 16–30 years, a subset of respondents in the 2016 Canada Food Study (CFS).

Beta coefficients are presented as incident rate ratios (IRRs), with 90% confidence intervals (CIs).

AIC, Akaike information criterion.

The reference category of sex is female.

The reference category of cities is Toronto.

Time-weighted ratio of fast food outlets in buffer of activity locations is the time-weighted ratio of fast food outlets to supermarkets, convenience outlets, fast foods outlets, and green grocery outlets combined in buffer of activity locations of an individual.

The time-weighted density of built environment is the time-weighted sum of ALE_TRANSIT (Active Living Environment Index with transit z score included) in the dissemination areas (DAs) containing the activity locations of an individual. Derived from Canadian Active Living Environments Database (Can-ALE) 2016, ALE_TRANSIT is the sum of the z-score of the intersection density, dwelling density, points of interest, and transit measures [1].

The temporal weight is the proportion of time spent in each of the activity locations relative to the total time spent in all activity locations of an individual.

*P<0.1, **P<0.05, ***P<0.01.

## **References**

1. Ross N, Wasfi R, Herrmann T, Gleckner W. Canadian Active Living Environments Database (Can-ALE) [Internet]. Canada; 2016 [cited 2018 Nov 30]. Available from: https://nancyrossresearchgroup.ca/research/can-ale/
